# Inherited Deletion of 1q, Hyperparathyroidism and Signs of Y-chromosomal Influence in a Patient with Turner Syndrome

**DOI:** 10.4274/jcrpe.galenos.2018.2018.0005

**Published:** 2019-02-20

**Authors:** Alejandro F. Siller, Alex Shimony, Marwan Shinawi, Ina Amarillo, Louis P. Dehner, Katherine Semenkovich, Ana María Arbeláez

**Affiliations:** 1 Washington University Faculty of Medicine, Department of Pediatrics, Washington, USA; 2 Washington University Faculty of Medicine, Department of Pathology and Immunology, Washington, USA

**Keywords:** Turner syndrome, genetic testing, hyperparathyroidism, inherited 1q deletion, signs of Y-chromosomal influence

## Abstract

We report a detailed phenotypic, cytogenetic and molecular characterization of a patient prenatally diagnosed with Turner syndrome (TS). In addition to having typical TS clinical characteristics including webbed neck, high arched palate and coarctation of the aorta, the patient had features less frequently seen in TS. These included recurrent parathyroid adenomas, growth along the 75^th^-90^th^ centiles on the TS height curve despite minimal treatment with growth hormone, behavioral problems and evidence of gonadal dysgenesis with testicular-like structures, such as seminiferous tubules lined by Sertoli cells and a contiguous nodule of Leydig cells. While fluorescence in situ hybridization (FISH) failed to detect Y-chromosome material in gonadal tissue or blood samples, chromosomal microarray analysis (CMA) confirmed X monosomy and a 4.69 Mb copy number loss on 1q31.2q31.3 (bp 192,715,814 to 197,401,180). This region contains the *CDC73* gene which has been associated with hyperparathyroidism-jaw tumor syndrome, features of which include recurrent, functional parathyroid adenomas and behavioral issues. This case illustrates how atypical features in a TS patient, such as robust growth and recurrent parathyroid adenomas, may suggest an underlying molecular etiology that should be explored by additional genetic diagnostic modalities. It is therefore appropriate in such cases to conduct further genetic testing, such as CMA and FISH, to explore other diagnostic possibilities and possibly prevent further complications.

**What is already known on this topic?**

Turner syndrome (TS) is caused by partial or complete absence of a second sex chromosome resulting in phenotypes involving multiple organ systems. Endocrine problems typically manifest as short stature, gonadal failure and hypothyroidism. Hyperparathyroidism, though reported in a small number of TS cases, is not considered a typical feature.

**What this study adds?**

This case highlights the importance of genetic testing beyond karyotype in patients with atypical clinical features of Turner syndrome (TS), particularly in those with hyperparathyroidism (HPT). The findings also led us to postulate a potential molecular mechanism for HPT in prior TS cases.

## Introduction

Turner syndrome (TS) is a disorder caused by partial or complete absence of a second sex chromosome and results in a wide variety of phenotypes with an estimated incidence of 1/2000-1/3000 live births (1,2,3). Certain features, such as short stature and primary ovarian failure, are present in virtually all cases. Coarctation of the aorta, bicuspid aortic valve, renal anomalies, autoimmune thyroid disease and hearing loss are also frequently present in patients with TS. While rare, hyperparathyroidism (HPT) has been reported in a small number of TS patients (4,5,6,7,8). Further genetic testing in these cases was not performed, thus the etiology of these concurrent morbidities is unknown. Other reported atypical clinical features have been suggestive of other clinically relevant genomic abnormalities (9,10). 

Herein, we present a case of TS with HPT who was found to have a 1q deletion. Patients with 1q deletions have been reported to have growth retardation, psychomotor delay and genital, cardiac and facial anomalies as well extremity anomalies (11). Additionally, *de novo* deletions in this region have been associated with developmental delay, agenesis of the corpus callosum and cerebellar hypoplasia (12). Moreover, this region includes genes that have been associated with recurrent, functional parathyroid adenomas as well as behavioral issues. While HPT has been shown to be associated with both 1q deletions (13) and TS separately, to our knowledge HPT has not been documented in an individual patient with both a 1q deletion and TS. The findings in our patient suggest a possible genetic cause, beyond the missing sex chromosome, of other TS patients exhibiting these types of atypical clinical features and highlights the importance of a multidisciplinary approach and genetic testing, beyond karyotyping, in atypical TS cases.

## Case Report

The proband was a 20-year-old woman with classical features of TS, including webbed neck, widely-spaced nipples, a high-arched palate, a bicuspid aortic valve, coarctation of the aorta (surgically repaired at one year of age) and a 45,X karyotype on an antenatal amniocentesis. Other comorbidities included bipolar disorder, dyscalculia, bilateral kidney malrotation, steatohepatitis and an episode of hemorrhagic gastritis of unclear etiology. At age 11, she was found to have an elevated plasma calcium level of 12.1 mg/dL [reference range (RR): 8.5-10.3 mg/dL], an intact parathyroid hormone (PTH) level of 369 pg/mL (RR: 14-72 pg/mL), a plasma phosphorus level of 1.7 mg/dL (RR: 3.0-6.0 mg/dL) and a urinary calcium to urinary creatinine ratio of 0.19. Technetium-99m (Tc-99m) sestamibi scan revealed an enlarged right superior parathyroid gland. She underwent resection of the enlarged parathyroid and surgical pathology showed a right superior parathyroid adenoma measuring 1.1x1.0x1.6 cm and weighing 1.07 grammes. Intraoperative sampling of the right internal jugular vein showed a drop in PTH from 815 to 42 pg/mL following resection. Five months post-surgery, she developed abdominal pain and emesis and was found to have a left distal ureteral calculus, left hydronephrosis and bilateral nephrocalcinosis and bilateral nephrolithiasis, leading to a ureteroscopy with stone extraction. At that time her urinary calcium to urinary creatinine ratio was 0.12. Post-stone extraction, she remained normocalcemic until age 16, when she was found to have an elevated plasma calcium level of 11.4 mg/dL, elevated intact PTH level of 108 pg/mL and a plasma phosphorus level of 3.8 mg/dL. Neck ultrasound showed a solid, hypoechoic nodule posterior to the midportion of the right thyroid measuring 9x6x4 mm with detectable internal vascularity on Doppler, consistent with a second enlarged parathyroid. The Tc-99m sestamibi scan did not show an area of increased activity, but given ultrasound findings and biochemical results she had a second parathyroidectomy, yielding a 0.136 gramme, hypercellular parathyroid and a decrease of the intraoperative PTH from 136 to 28 pg/mL. She has been normocalcemic since.

**Figure 1:**
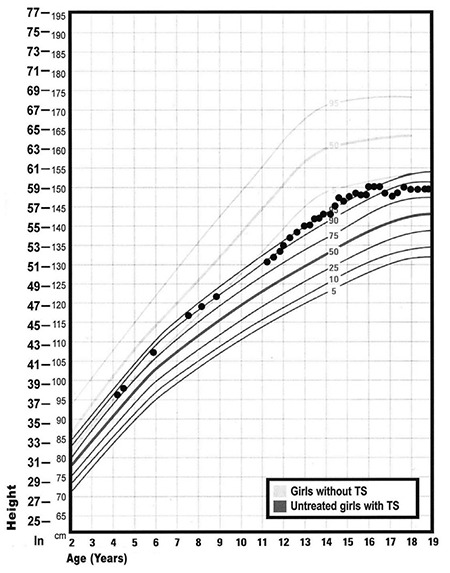
Proband’s growth points on growth chart for children with Turners syndrome (Adapted from Frías JL, Davenport ML; Committee on Genetics and Section on Endocrinology. Health supervision for children with Turner syndrome. Pediatrics 2003;111:692-702) TS: Turners syndrome

The patient grew along the 75^th^-90^th^ percentiles of the TS height-for-age growth chart (14) since birth. Her final height prediction, given her parental heights, was 171 cm. Growth hormone therapy (0.35 mg/kg/week) was initiated at seven years of age. However, her family felt this treatment led to agitation and overactivity and was therefore discontinued after less than one year of therapy. It was never restarted and she continued to grow along the 90^th^ percentile for TS, achieving an adult height of 150 cm, consistent with roughly the 1st percentile of the CDC growth chart for girls without TS (Figure 1) (14).

The proband required special education classes for learning disabilities, especially in mathematics which is typical of girls with TS, and was also diagnosed with attention-deficit/hyperactivity disorder. Last audiogram at age 20, revealed mild left ear hearing loss at 4-8 kHz and mild right ear conductive hearing loss from 250-8 kHz. The proband can do most of her daily life activities without any help.

Verbal informed consent was obtained from the patient and the family.

**Figure 2:**
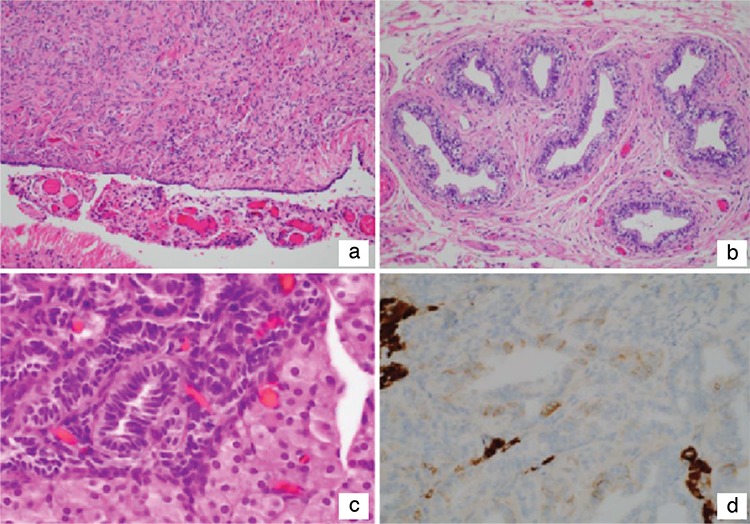
Histopathological findings of gonadal tissue. (a) The right (shown) and left gonads show the typical fibrous, ovarianlike stromal features of the streak gonad. (b) This circumscribed nodule is composed of epithelial-lined tubules surrounded by fibrous stroma with a resemblance to the epididymis. (c) The tubular structures resembling Sertoli tubules in the absence of germ cells are adjacent to a nodule of Leydig cells with abundant pale eosinophilic cytoplasm. (d) Inhibin immunostain shows pale staining of the tubules and intense reactivity in the Leydig cells

She had documented primary ovarian failure at age 14 with elevated gonadotropins (luteinizing hormone: 18.9 IU/L and follicle-stimulating hormone: 99.8 IU/L) levels. Gradual estrogen replacement therapy with conjugated estrogen was started at that time and she experienced menarche a year later. She then began combined oral contraceptive therapy (OCT), but developed severe mood-related symptoms and extreme distress from breakthrough bleeding that required treatment with multiple mood stabilizing medications (Prozac, Zyprexa, Lithium, Seroquel). The progesterone in her OCT was felt to be the primary trigger for this exacerbation in her mood symptoms. Thus, she elected to undergo a hysterectomy with bilateral salpingo-oophorectomy (BSO) at age 18 in order to resume estrogen-only therapy. Following hysterectomy/BSO, the patient was continued on estrogen-only replacement with improvement of mood disturbance. The pathology showed a diminutive uterus weighing 33 grammes. The bilateral adnexa had fallopian tubes and fibrous streak gonads. In addition, the right-side streak gonad (Figure 2a) was accompanied microscopically by ovarian-like stroma, dysgenetic testicular-like structures and an apparent vas deferens. The right gonad showed the presence of a fibroepithelial structure with the features of an epididymis (Figure 2b) and a second nodule composed of Sertoli-like tubules with an adjacent focus of Leydig cells (Figure 2c). Inhibin immunostain confirmed the presence of Leydig cells (Figure 2d). The patient had never had any physical examination findings suggestive of virilization.

**Figure 3:**
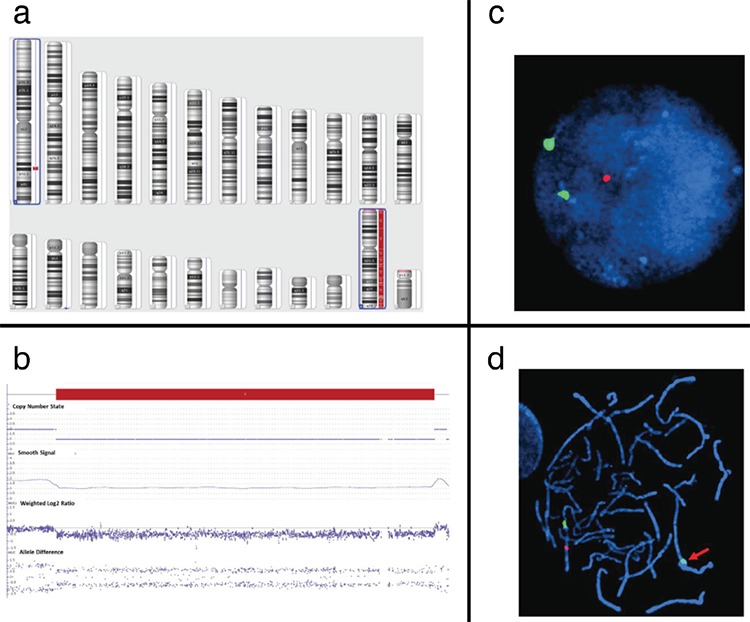
Proband CMA: (a) Karyoview showing concurrent copy number losses (blue boxes) on chromosomes X (monosomy X) and 1 (1q31.2q31.3; ~4.69 Mb, 4,113 markers/probes, arr[hg19] 1q31.2q31.3(192715814-197401180)x1 mat). (b) Detailed view of the 1q31.2q31.3 deletion (CN=1, smooth signal=1, weighted log2 ration=-0.5, allele difference=+0.5, -0.5). Post- CMA familial FISH: (c) Interphase and (d) metaphase proband and maternal cells revealed one copy number loss CMA: chromosomal microarray analysis, FISH: fluorescence in situ hybridization

Since the patient presented with atypical features of TS, including HPT, an unusual growth pattern, behavioral abnormalities and the presence of gonadal dysgenesis with Sertoli-only tubules, endocrinology recommended that the genetics team become involved. Thus both a chromosomal microarray analysis (CMA) of the proband’s peripheral blood and a fluorescence *in-situ* hybridization (FISH) analysis of the peripheral blood and of the testis-like structures in the streak gonad tissue were performed. The Affymetrix CytoScan HD (www.affymetrix.com) was utilized to interrogate the genomic DNA for copy number variants (CNVs) and regions of homozygosity (ROH). The array was designed with 2.6 million copy number markers, including 1.9 million non-polymorphic probes, selected for their linear response to copy number and genomic position. The average intragenic marker spacing is equivalent to 1 probe per 880 base pairs. A genomic imbalance is reported when deletions are greater than 200 kb and duplications are greater than 500 kb, unless they represent a region clearly associated with benign copy number polymorphism in multiple independent studies. ROH are reported when they are greater than 10 Mb. The genomic linear positions are given relative to GRCh37/hg19 (UCSC Genome Browser) (15). Copy number analysis was done using the Affymetrix Chromosome Analysis Suite (version 3.0.0.42 r8004). The CMA of the proband revealed two CNV: a loss of the entire chromosome X (~155 Mb) indicative of monosomy X and a 4.69 Mb copy number loss on 1q31.2q31.3 (bp 192,715,814 to 197,401,180) (Figure 3).

Interphase and metaphase FISH analyses on peripheral blood lymphocytes, obtained from the patient and her parents, were performed using standard cytogenetic methods, to confirm the 1q deletion in the proband, which was also found to be maternally inherited. The RP11-78E12 BAC clone and CEP 1 FISH probes (Empire Genomics LLC, Williamsburg NY) were used to detect the 1q deletion and centromere 1 (control) regions, respectively. 

Interphase *SRY*/Y FISH was also performed on paraffin-embedded tissue obtained from the testicular-like structures in the dysgenic right gonad with locus-specific Vysis commercial FISH probes localizing to centromere X (CEPX; DXZ1; Xp11.1-q11.1 Alpha satellite DNA; Spectrum Green) and sex-determining region Y (SRY; Yp11.31-p11.32; Spectrum Aqua) and Yq12 Satellite III DNA locus (DYZ1; Spectrum Orange (Abbott Molecular, Des Plaines, IL). The testicular-like structures showed a single X signal pattern. None of the nuclei showed the presence of* SRY* or Yq-specific signals such as *DYZ1*. The tubular structures were weakly positive for *WT-1*, but *SALL4* was non-reactive indicating an absence of germ cells in the tubules (not shown).

## Discussion

Awareness of the typical features of TS is important in order to diagnose this disorder as early as possible and treat any associated comorbidity. There are studies showing that the recognition of TS is occurring earlier, with serial surveys in Belgium suggesting that the age at diagnosis, especially TS with a 45,X genotype, declined from 11.2 years of age in 1991 to 6.6 years of age in 2003 (16). Moreover, it is crucial for clinicians to be aware of atypical aspects of a TS patient’s presentation and to pursue further genetic testing if necessary. In addition multidisciplinary involvement in such cases will help to mitigate the risk posed by these atypical manifestations.

Primary HPT has been reported in a small number of TS cases (4), but to our knowledge, there have been no studies investigating the etiology of primary HPT in patients with TS. HPT-jaw tumor (JT) syndrome, which is associated with recurrent functional parathyroid adenomas as well as behavioral issues, has been shown to be associated with a large-scale 1q31 deletion, specifically the *CDC73* gene (13). CMA in our patient revealed a maternally inherited 4.69 Mb deletion at 1q31.2q31.3. Interestingly, the deleted interval in this patient includes 16 OMIM genes, including *CDC73* gene, also known as *HPRT2*. Loss of function mutations in *HPRT2* have been associated with HPT, parathyroid adenoma, parathyroid carcinoma or HPT-JT (17). *HPRT2*, which encodes parafibromin, is a regulator of gene expression through its association with the RNA polymerase II subunit *POLR2A* and with a histone methyltransferase complex (18). In eight previously documented cases of 1q deletions encompassing the 1q31.2q31.3 region, most presented with growth retardation, psychomotor retardation, and lip/palate anomaly phenotypes. In our patient, her bipolar disorder, behavioral abnormalities, learning disabilities and atypical aspects might be associated with inherited deletion of 1q (11). The patient’s parental FISH revealed that the proband’s mother is a carrier for the same 1q31.2-31.3 deletion, but she has no clinical or laboratory evidence of HPT. This may be due to incomplete penetrance or phenotypic variability. However, she will warrant surveillance for calcium and parathyroid abnormalities in the future.

There are currently no consensus guidelines for monitoring calcium and/or PTH levels in patients with *CDC73* related conditions. One report suggests screening for HPT in HPT-JT with serum calcium and PTH levels every 6-12 months (19), similar to predisposing syndromes for parathyroid tumors, such as multiple endocrine neoplasia syndrome type 1 (20).

Relative height stature has been reported in TS patients with Y chromosome material or with karyotypes other than 45,X (21). A wide phenotypic variability in mixed gonadal dysgenesis has been previously described, with unilateral testicular structures, due in part to isodicentric Y(p) (idicY(p)) mosaicism or the presence of *SRY* in early gonadal ontogenesis of Sertoli cells (22). We report the phenotypic and cytogenetic characterization of an apparently female patient, with mixed gonadal dysgenesis who unexpectedly was found to have a histologically male gonad with Sertoli cells. However, despite histopathological evidence of Sertoli cells in our proband, FISH analyses of peripheral blood and gonadal tissue did not demonstrate evidence of *SRY* material or idicY(p) mosaicism, suggesting that additional factors may play a role in gonadal determination and differentiation, such as the timing of the mitotic loss of the Y material during gonadal ontogenesis and the proportion of *SRY* positive pre-Sertoli cells in the gonad. TS patients with presence of *SRY* or Y-chromosome material have a 7-33% risk of developing gonadoblastoma (23,24,25,26). Therefore, given the pathologic findings in the right gonad, the risk of gonadoblastoma in this patient was not insignificant (regardless of whether or not we were able to find evidence of Y-chromosome material) and it was addressed with surgery. It is important to note, however, that it can be difficult to locate Y-chromosome material even in those with clear evidence of developmental influence of *SRY*, as seen in the proband in this report. Further research is needed to determine the risk of gonadoblastoma in patients with testicular tissue but with negative *SRY* on peripheral blood and gonadal tissue.

While classical cases of TS with 45,X karyotype and typical features of neonatal pedal edema, short stature, ovarian failure and cardiac comorbidities are frequently diagnosed earlier by discerning physicians, atypical features warrant investigation beyond the conventional karyotype study. CMA analysis continues to improve the detection of additional chromosomal aberrations in patients with TS and facilitates genotype-phenotype correlation. Such clinically significant information can lead to additional interventions, such as gonadectomy to eliminate the risk of gonadoblastoma in the presence of cryptic Y-chromosome material or establish surveillance for other complications. In this case, the proband’s height, recurrent parathyroid adenoma, behavioral problems and learning disabilities warranted CMA, which led to the discovery of a 1q31.2q31.3 deletion. The male-differentiated structures in one streak gonad suggested the influence of SRY during gonad development, which ultimately led to gonadectomy. Since this may in fact be the first documented case of TS, HPT and a large 1q deletion, it should prompt further evaluation of patients exhibiting phenotypes that may not be attributed simply to TS. Therefore, it is crucial for clinicians to be aware of the typical TS phenotype and pursue careful examination and further genetic diagnostic investigation in cases with unusual phenotypic features or less common co-morbidities. This will facilitate better understanding of the underlying molecular etiologies and genotype-phenotype correlation in atypical cases of TS. Such clinically significant information can lead to additional interventions and better patient surveillance to prevent complications.
